# Insanity, Disability and Responsibility: Rethinking Autonomy to Challenge Structural Inequality

**DOI:** 10.1093/ojls/gqae020

**Published:** 2024-06-19

**Authors:** Jane Richards

**Keywords:** CRPD, insanity, disability, autonomy, criminal responsibility

## Abstract

The Convention on the Rights of Persons with Disabilities (CRPD) operates as a lens of analysis to show that the insanity doctrine and its dispositions discriminate against the category of people with mental disabilities to whom the defence applies. However, while identifying the discrimination perpetuated by the insanity doctrine, this article argues that the CRPD Committee has failed to uncover the ultimate source of disadvantage of which the doctrine is merely symptomatic. Instead, it is argued that the criminal justice system entrenches a notion of ‘capacity-responsibility’ which situates the mentally disabled defendant as the ‘other’. In an attempt to challenge this embedded structural injustice, the article thus calls on the CRPD Committee for a more holistic application of the CRPD, to provide the tools to challenge that will move towards greater equality for people with mental disabilities.

## 1. Introduction

Consider the very ‘typical’ insanity case of *HKSAR v Lau Kam Fai*.^1^ The defendant had schizophrenia, which caused delusion beliefs. Believing himself to be warding away evil spirits, the defendant stabbed both his wife and his daughter.^2^ In the period leading up to the incident, the defendant’s mental health had rapidly deteriorated, two psychiatrists having diagnosed him with experiencing disordered thinking and hallucinations. The judge found that ‘On the day of the crime, his condition was quite serious, and he was completely unable to control himself at the moment of the crime’.^3^ There was no question that at the moment that the crime was committed, the defendant intended the harm that he caused. There was also no question that his acts were compelled by anything other than the onset of disease; his state of mind was completely out of character,^4^ and it was rare for him to lose his temper.^5^

Lau was granted an insanity acquittal under Hong Kong’s common law system. Deemed not criminally responsible,^6^ the judge made a 12-month hospital order for treatment in Siu Lam Psychiatric Hospital.^7^ On the basis of his mental disability, Lau was treated differently to other defendants who have been found guilty. In addition to being subject to a special defence which denied his legal capacity to act, in hospital, he would have been subject to coercive medical interventions, regardless of whether or not he consented to treatment. His period of detention and release would be made contingent on him proving that he no longer posed a threat to the community. This is a standard for release which almost all other offenders do not have to meet.^8^

It is on the basis of these rights violations, and the denial of the right to equality before the law, that the Committee for the Convention on the Rights of Persons with Disabilities (CRPD) has called for the reform or abolition of the insanity defence and similar non-responsibility doctrines and dispositions.^9^ Indeed, there is some consensus between both disability scholars and criminal law reformers that the insanity defence discriminates against people with mental disabilities.^10^ But whereas criminal lawyers seem resigned to working with a largely problematic defence,^11^ the CRPD Committee and some critical disability scholars have called for the universalisation of doctrines to make them ‘disability neutral’.^12^ This would mean either abolishing or reforming criminal law doctrines—such as insanity—to be replaced with doctrines that apply equally to all, regardless of disability. It is not clear whether disability would be rendered irrelevant to subsequent disposition orders; however, on this view, there is no scope for defences and dispositions which are disability specific.

There is a tension in balancing the public’s right to be protected from egregious violence and the rights of individuals with mental disorders to be properly supported for their inclusion in the community. The resulting debates in law, policy and scholarship provoke highly emotive, politically charged and often uncompromising perspectives, producing a clash in the drive for how these rights should be balanced and protected. And yet, despite the topical nature of these human rights issues, they have yet to be explored in depth in the literature. Proceeding from existing doctrinal and socio-legal uncertainty over how best to balance these seeming competing rights and priorities, this article breaks new ground by bringing these divergent perspectives to the table, and in doing so it identifies the ‘blind spots’ within existing debates;^13^ from the perspective of the CRPD, criminal law has paid insufficient regard to the rights of people with mental disabilities, and there is a failure to address the harm and stigma perpetuated by maintaining separate mental disorder defences.^14^ A minority of disability scholars have acknowledged the inherent complexities of reform within the existing criminal justice framework.^15^ However, the CRPD Committee and a significant number of other disability scholars have seemingly given little consideration to the legitimate goals of the criminal justice system in preventing crime and facilitating the rehabilitation of defendants whose disability has manifested in egregious crimes.^16^

I argue that while the insanity defence and its disposition orders *are* wholly unacceptable from a disability rights perspective, the reform solution offered by the CRPD Committee is neither viable nor realistic. I argue that the criminal justice system is inherently ableist, and it is this embedded ableism that is the ultimate source of oppression of people with mental disabilities. The insanity doctrine is merely symptomatic of this structural injustice, and in making doctrine the target for reform, the CRPD Committee’s rights-based ‘disability neutral’ approach does nothing to challenge these underlying norms.

In making this argument, I will return to the case of *Lau* and comparable insanity cases from Hong Kong and Canada. These cases illustrate the real and significant rights violations that people with mental disabilities face as a result of disability-specific doctrines and dispositions. These jurisdictions offer interesting materials for analysis; despite diverging somewhat in the robustness of human rights protections, both maintain a largely similar variation of the insanity defence—the substance remaining little changed since its initial inception in the *M’Naghten* case^17^ of 1843. Still, this article does not offer a comparative analysis, nor is it contingent on these specific examples. The real significance lies in the striking similarities in the way that law has evolved both in these and comparative jurisdictions; these same principles are reflected across the common law world and even in some civil law jurisdictions.^18^ As such, the analysis is broadly transferrable beyond these specific cases and jurisdictions.^19^

The discussion unfolds in four key parts (sections 2–5). In section 2, I introduce the insanity defence and its attendant disposition orders, to show the ways that it perpetuates discrimination against the category of mentally disordered defendants to whom it applies. Fundamentally, by denying that a defendant had the mental capacity to be held responsible for their conduct, the defendant is denied the right to equal legal capacity. This section lays the groundwork for section 3, which zeroes in on the way that within the institution of criminal justice, the notion of responsibility is constructed to set a baseline for normative functioning. It is in carving out the terrain of this responsible, legal subject that the criminal justice system situates the mentally disabled defendant as the ‘other’. In section 4, I return to the CRPD Committee’s challenge to the insanity doctrine. I argue that bringing equality for people with disabilities will not be achieved by targeting the doctrines that are the outward manifestation of organising institutional norms. In section 5, I thus call on the Committee to adopt a more nuanced approach in seeking reform. In doing so, I advocate for a shift away from a narrow focus on legal capacity towards a more holistic interpretation of the CRPD. Unfortunately, I am unable to offer a concrete alternative to the existing criminal justice system which would solve all the problems.^20^ Still, there is value in considering this specific criticism of the Committee’s interpretation within the broader context of the ableism embedded within the criminal justice system. In the pursuit of equality and non-discrimination for people with mental disabilities, I create space for the discussion of reform which aims to tackle the underlying assumptions and norms that entrench discrimination against this category of people within the criminal justice system.

The aim of the article is to facilitate a long overdue discussion to open the door for meaningful and viable future reform. In the spirit of the CRPD, my aim is to bring recognition of the structural oppression that is embedded within the institution of the criminal justice system against people with mental disabilities. In making this argument, the article attempts to move beyond contemporaneous debates which position disability rights somewhat in conflict with the priorities of the criminal law, making space to challenge the ableist structural oppression embedded within the criminal justice system.

## 2. Background: Discrimination Perpetuated by the Insanity Defence through a CRPD Lens of Analysis

### A. The Erosion of Rights: From Trial to Disposition

It is striking that much consensus can be found between criminal lawyers and a significant number of disability scholars that insanity and its disposition orders perpetuate discrimination against people with mental disabilities.^21^ Broadly, criminal lawyers emphasise the need to balance ‘the interests of the individual defendant and to the public risk which he represents’.^22^ In striking this balance, the emphasis has fallen in favour of the protection of the public from harm. In this sense, the inherent problems with the defence are outweighed by the difficulties of drastic reform.^23^ However, the CRPD Committee takes issue with this point, and has said that insanity is completely incompatible with the right to equality before the law for people with mental disabilities, calling instead for its abolition.^24^

The social model of disability embedded in the CRPD sees barriers which are external to the person as the root cause of disability discrimination against people with impairments. To achieve equality, those barriers must be removed by reform or abolition. The insanity defence is an example of such a barrier to equality; at the pre-trail, trial and disposition stages, it applies specifically by reason of disability, setting off a chain of rights violations which erode fundamental tenements of personhood.^25^

This is best understood by reference to the specific rights which are guaranteed in the CRPD, viewed in light of criminal procedure and domestic case law. For example, in Hong Kong, a lawyer may refuse instruction from a client whom they perceive has a mental disability.^26^ Similarly, the Hong Kong Solicitors’ Guide to Professional Conduct provides that a solicitor may not allow their client’s wishes to override their professional judgment.^27^ Instead, counsel’s impression of their client’s best interests is to take precedence over their client’s wishes.^28^

At the trial stage, when mental disorder is raised, the balance struck between the defendant’s right to a fair trial and the potential risks of violence posed by defendants has consistently been weighed in favour of protection of the public. Canada’s Supreme Court considered its constitutional guarantees to equality^29^ in the context of insanity in the case of *R v Swain*.^30^ The issue was whether the defendant had an absolute right to control his trial. The Court held that once the defendant puts forward evidence which brings their mental capacity into question, the trial judge is entitled to put the matter of insanity to the jury. The Court held that the Charter of Rights and Freedoms would not be breached where the prosecution wished to raise evidence of insanity over the wishes of the defendant. In balancing the principles of fundamental justice against the public’s right to be free from the threat of violence, the Court held that enabling the prosecution to raise evidence of insanity is a reasonable limit on the defence’s right to control their own trial.

The Hong Kong courts go further in eroding a defendant’s right to control their own trial. In Hong Kong, not only may prosecution raise the insanity defence against the defendant’s wishes, but defendant’s counsel may have an *obligation* to do so if they assess it to be in the defendant’s ‘best interests’.^31^ In *HKSAR v Chow Kin Chung*,^32^ the court held that:

If the nature of a defendant’s criminal record and counsel’s submissions indicate a chronic mental health problem, it behoves the court to examine the matter, *regardless of whether the Applicant has relied on it*, or whether counsel has asked the court to investigate further. If defence counsel does not suggest that a psychiatrist report be obtained, it is open to the judge to obtain one before sentencing, particularly if the circumstances of the offence are somewhat unusual. Further, if what the Applicant needs is a Hospital Order and such an order is recommended, it is usually *not desirable that the judge ignores such an order* as the best sentencing option.^33^

This approach embeds a medical model of disability, where significant deference is shown to medical experts, eroding the rights to a fair trial in a way that does not apply to non-disabled defendants.^34^

Insanity dispositions constituting hospital orders trigger serious rights violations, and the case of *Barker v Barker*^35^ is an extreme example of this. The defendants in that case had been found not guilty by reason of mental disorder. Under the guise of therapeutic intervention, the complainants in that case were subjected to treatment so degrading that the judge described it as torture.^36^ In terms of orders for release, in the Canadian Supreme Court case of *Winko v British Columbia (Forensic Psychiatric Institute)*,^37^ the Supreme Court held that the ‘paramount consideration’ for the relevant review board is the ‘safety of the public’; other considerations, such as the defendant’s mental health, the reintegration of the accused into society and the accused’s other needs, are secondary.^38^

At each stage of the process, people with mental disabilities to whom the insanity defence applies are at risk of their rights being denied. Denying access to counsel erodes equal access to justice.^39^ Limiting rights to a fair trial breaches the right to be recognised as equal before the law.^40^ Hospital orders which are ordered on the basis of disability violate the right to liberty.^41^ Medical treatment that is non-consensual breaches rights to personal integrity,^42^ and may be understood to constitute abuse^43^ and even torture.^44^

### B. A Denial of Equal Legal Capacity

Running throughout these rights violations is the denial of legal capacity. Article 12, which has become perhaps the most debated and significant article in the CRPD, guarantees the right to equal recognition before the law. In General Comment 1, the CRPD Committee has made clear that Article 12 ‘is operative “everywhere”’^45^, and that ‘there are no permissible circumstances under international human rights law in which a person may be deprived of the right to recognition as a person before the law, or in which this right may be limited’.^46^ The CRPD Committee has said that legal capacity is a ‘universal attribute inherent in all persons by virtue of their humanity’,^47^ and that people with disabilities have the right to ‘enjoy legal capacity on an equal basis with others in all aspects of life’.^48^ General Comment 1 also says:

Legal capacity means that all people, including persons with disabilities, have *legal standing* and *legal agency* simply by virtue of being human. Therefore, both strands of legal capacity must be recognized for the right to legal capacity to be fulfilled; they cannot be separated.^49^

However, the insanity defence exists in complete conflict with the Committee’s interpretation. Insanity denies that people to whom the defence applies have the legal standing or legal agency to be held responsible for their actions. The insanity defence operates to deny that a person is autonomous, to find that their mental capacity was so impaired as to render them not criminally responsible on the basis of disability. Defendants to whom the defence applies are not held to the same standards of accountability as all other offenders. In other words, because people with mental disabilities in this category are assessed to lack the mental capacity for criminal responsibility, their legal capacity is denied.

On the Committee’s view, legal capacity is a separate and distinct concept from metal capacity.^50^ Differences in mental functioning are part of the variation of what it is to be human.^51^ Respect for equal legal capacity requires the recognition of the equal worth of all variations of mental capacity, to ensure that all people are respected as autonomous agents, regardless of impairments in mental functioning.^52^ Mental incapacity can never be used as grounds to deny legal capacity.^53^

Respect for autonomy extends to respecting decisions made by the person, irrespective of whether a person’s decision was animated by mental disability. It is a violation of the right to equality before the law to deem some decisions as less worthy of legal recognition than others on the basis of disability. This is not to say that people with disabilities do not make bad decisions, including decisions which may be in breach of criminal laws.^54^ But, given that all *other* people are free to make mistakes and bad decisions,^55^ respect for the autonomy of the person requires decisions by people with mental disabilities to be respected. People with mental disabilities must be held to account on an equal basis with all others.^56^ This is a robust interpretation of legal capacity,^57^ which demands the ‘levelling up’ of respect for all variations of mental functioning. Disability-specific doctrines which deny that a decision is autonomously made thus abrogate the right to equality before the law.

In conflict with the Committee’s interpretation of equal legal capacity, the insanity defence *does* distinguish between the quality of decision-making abilities. Insanity is premised on the notion that some people are not truly autonomous because their decision-making abilities are so severely impaired by disability that they are not in control of their decision-making processes, able to reason objectively, rationally respond to their circumstances or comprehend reality in accordance with normative standards. The insanity defence assesses that the person lacks the requisite mental capacity to be attributed legal capacity. The person’s severe mental impairment abrogates responsibility for decisions made, and the defendant is acquitted of legal and moral blame.^58^ In the next section, I draw on criminal legal theory to show the expressive implications of this demarcation between the autonomous subject who is answerable for their conduct and the mentally disabled defendant.

## 3. Disability as the ‘Other’

### A. The Responsible Subject in Criminal Law

Across both common law and civil law jurisdictions, some variation of insanity exists to exculpate and divert from punishment^59^ defendants who are assessed to lack the mental capacity to be autonomous. Broadly, the presumption of sanity operates as a threshold for the attribution of criminal responsibility.^60^ As described by Lindsay Farmer, the criminal justice system assumes a ‘certain kind of responsible agency: persons are *capable* of being guided by norms and may accordingly be answerable for their conduct when they breach those norms’.^61^ In Nicola Lacey’s words, in the modern criminal law, ‘the rational for conviction and punishment … is founded on a particular understanding of the defendant’s status as a moral agent: a reasoning being responsible for his or her beliefs, desires, emotions and values’.^62^

Ordinarily, legal subjects are assumed responsible for their actions. Where the minimum standards of civility set out by the criminal law are breached, the person will be held responsible for that criminal conduct.^63^ Farmer writes: ‘The responsible individual is thus central to the modern normative imagination, shaping accounts of the rights and obligations that individuals owe to each other and understandings of the relationship between individuals and the state.’^64^

In this sense, criminal responsibility is *qualitative*; it comprises specific capabilities, such as rationality, reason and an ability to be responsive. While these capabilities are presumed, they are not universal. Rather, the presumption of sanity sets the minimum threshold for a person to be held to be legally and morally responsible for their actions. Autonomous personhood is constructed on assumptions that the person is capable of understanding civic norms and has the capacity to shape their conduct in a way that complies with those norms. Nicola Lacey thus describes responsibility as being ‘founded in capacity’.^65^ Of this notion of ‘capacity-responsibility’, she writes:

Capacity responsibility makes a strong assumption about what it is that we are responsible for: we are responsible not for our selves, for who we are, or for our social status, but—on a quasi-contractual basis—for the specific acts which we (choose to) do or (in limited circumstances) refrain from doing. Under this notion of capacity responsibility, respect for agency and individual freedom is central … Only when criminal law is addressed to human beings as choosing subjects capable of conforming their actions to the criminal law can it be compatible with individual freedom.^66^

To be held responsible, a person must have the mental capacity to be answerable for their conduct. The presumption of sanity is the bright line which divides those who meet minimum standards of mental functioning from those who fall below. But the presumption of sanity does more than divide two categories of defendants on the basis of mental functioning. Expressively, it *constructs* the responsible, normative individual in law, segregated from the insane, to whom the usual norms of functioning and accountability do not apply. From the perspective of maintaining civil order and restoring normative, ie autonomous functioning, the criminal justice system thus compels differential treatment.

### B. The Elements of Proof and the ‘Terrain of Mental Incapacity’^67^

There is a divergence between the ‘physical’ and ‘fault’ elements^68^ of an offence, both of which must be proved. A guilty verdict requires proof of the intersection of the physical and mental elements of crime, that is, a coincidence of the *actus reus* and *mens rea*.^69^ An established nexus of these elements of proof establishing guilt triggers the classical principles of sentencing in criminal law—rehabilitation, deterrence, prevention and rehabilitation.^70^

In cases of insanity, rather than the usual *mens rea* requirement, there is coincidence of a ‘disease of the mind’ which is assessed to have compelled the physical elements of the crime.^71^ Broadly, insanity exculpates blame and diverts from punishment^72^ those accused for whom *mens rea* is entirely lacking or, alternatively, could not reasonably understand that their actions were wrong by reason of an animating mental disability.^73^ Rather than having relative freedom to make autonomous choices, the person’s mental disability constrained their decision-making abilities to the extent that they were prevented from being able to reasonably to avoid their criminal conduct. As a matter of proof, the person’s mental capabilities are judged to have disabled autonomy. The prosecution is still required to prove the physical elements^74^ of the crime; however, rather than the coincidence of an autonomous though guilty mind, the nexus is between a physical act and a mind that is wholly animated by mental disability.

The assessed absence of capacity compels a different type of analysis of the person’s mental state when compared with all other offenders. Ordinarily, whether a person is to be held criminally responsible for their actions is assessed at the moment the crime is committed. Taking a ‘time-slice’^75^ approach to assessing guilt, the defendant’s *mens rea* is assessed at the time of the *actus reus* to establish the defendant’s state of mind at the moment that the crime was committed. However, where the defendant’s mental disorder is in issue, mental disability becomes the lens of analysis^76^ through which a person’s range of choices, such as impulse control, the quality and speed of choices made, and whether a decision is rational is assessed.^77^ Mental incapacity may be evaluated as causative of conduct, and also comes to define the entire person. Dissimilar to other defendants, the analysis may also branch out to consider the defendant’s character, their history and their medical records.^78^ Fundamentally, the issue is whether the person has the ability to be held responsible by normative standards. The inability to respond to reason or understand reality is equated with unpredictability, increased risk and dangerousness.^79^ Applying the criminal law’s preventative and habilitative aims provides justification for the incapacitation and intervention^80^ which seek to contain the threat posed by this unpredictable ‘other’.^81^ Expressively, this category of defendant exists as a threat to the normative order,^82^ because they exist outside the bounds of the normative being who is capable of being ordered and responsive to social and legal norms.

It is because the person’s mental disability has manifested in violence that it is not able to be accommodated within usual norms of criminal law.^83^ This type of disability is unresponsive to principles of punishment and deterrence. From this perspective, limits on the rights and specific disposition orders are justified by the criminal law’s own internal logic, which seeks to maintain civil order.^84^ From the criminal law’s perspective, diversionary disposition orders which aim to return the person to a normative state of being provide a solution as to how to manage people with significantly impaired capacity.^85^ If the aim of rehabilitation is to restore autonomous functioning and enable reintegration of the person in the community, then coercive interventions which restore mental capacity may be justified. Rehabilitation aims to restore the status of full moral agent to make the person capable of following civil norms.

The segregation between the normative, punishable offender and the incapacious, undeterrable defendant has significant expressive implications. Mental disorder defences set specific categories of defendants apart as ‘qualitatively’ as opposed to ‘quantitatively’ different,^86^ and offenders in this category come to occupy the terrain of the ‘other’.^87^ These defendants represent a ‘different in kind’ as opposed to being ‘different in degree’.^88^ Historical stereotypes which see mental disability as the legitimate target of segregation, control, non-consensual treatment and even torture^89^ are reinforced, marking out the insane defendant as inherently flawed.

Those who lack mental capacity are rendered objects of the law, as opposed to equal subjects before the law. The structural result is to perpetuate ‘politics of difference’.^90^ Particular variations in mental functioning are assumed more dangerous than all others, unknowable, unpredictable and less than fully human.^91^ To the extent that responsibility is the ‘lynchpin’ of the modern criminal justice system,^92^ emphasis on this singular aspect of a person constructs and reinforces the ‘manifest criminality’^93^ of those who lack this fundamental quality which is required for recognition of full personhood.

Invisible barriers to inclusion operate to exclude this category of defendant from a robust notion of personhood. Disability disadvantage is not just reinforced, it debilitates.^94^ The disabled mind constitutes a diminished notion of personhood, and reinforces historical stigma that, as described by Linda Steele, the disabled individual is less worthy of rights protection because they have fewer rights to protect.^95^ Insanity is not a sort of accommodation; rather, it relegates, debilitates and politicises the mentally disabled mind.

By setting up disability as the ‘other’, insanity manifests in doctrine the structural oppression of people who cannot meet the criminal justice system’s constructed normative standards. In turn, by constructing the irresponsible other, insanity works to reinforce the privileged position of the capacious, responsible, legal subject.^96^ This constant refinement of normative standards of what it is to be an autonomous subject in law renders people with mental impairments as relatively powerless in how they are defined or how they come to interact with the criminal justice system.^97^ Severe mental disability is reduced to the subject of clinical diagnosis, and the criminal justice system’s doctrines and processes are the mechanisms of control applied to the bodies and minds of disabled defendants through benevolent plans of habilitation. The biopolitical construction of identity renders defendants in this category legitimate objects of state control. As argued by Linda Steele, this othering carves out mental disability as a political identity within the institution of criminal justice.^98^ It is my argument that in carving out a special space beyond the usual guilty and innocent dichotomy, the insanity defence is a product of, and also works to reinforce a particular conception of, responsibility and autonomy. To this end, responsibility and autonomy become synonymous with the normative, answerable subject in law.

## 4. The CRPD Committee’s Interpretation of Legal Capacity Applied to Insanity

### A. The Right to Equality before the Law

It is a key contention of this article that within an institution premised on presumptions of capacity, the norm of criminal responsibility structurally oppresses people with mental disorders, manifesting both in discrimination and the othering of disability. The CRPD Committee has recognised the way that the insanity defence manifests discrimination against the category of people to whom it applies.^99^ However, in its rights-based approach, the CRPD Committee see disability discrimination as a problem of doctrine, rather than understanding this doctrine as merely symptomatic of an institution which is inherently ableist in its construction. And while it is beyond the Committee’s mandate to institute large-scale reform of the criminal justice system, it *is* within its mandate to identify any barriers to inclusion which construct the disabled mind as the other.

Thus, despite making the case for the necessity of doctrinal reform,^100^ the CRPD Committee has not substantively engaged with the implications of reform. This means that even if insanity is abolished or reformed, without more, the underlying norms which shape the criminal justice system will remain intact. Premised on a ‘pure’ disability^101^ model, the CRPD Committee’s claims for equal rights are inherently limited to claiming rights which are available within existing institutions.^102^ The problem is that in calling for the equal recognition, or ‘levelling up’ of legal capacity, the CRPD Committee’s particular interpretation of legal capacity actually *reinforces* the criminal justice system’s underlying norms of capacity-responsibility. In other words, obfuscating the way that mental disability can be inherently disabling, while nonetheless calling for the ‘responsibilisation’ of mentally disabled defendants, reinforces the criminal justice system’s ableist normative framework.

### B. The CRPD’s Construction of Legal Capacity and the Implications for Decision Making

On the Committee’s interpretation, ‘“unsoundness of mind” (and other discriminatory labels) are not legitimate grounds to deny legal capacity’,^103^ because on its view, this constructs a limited and discriminatory conception of personhood.^104^ To this end, disability scholarship challenges the assumption of the normative individual in law who is an atomistic, self-reliant individual, who does not need support for decision making.^105^ It is discriminatory to draw distinctions between the different ways that different people make decisions, regardless of whether or not a decision is animated by a mental disability.

In drawing out the discrimination perpetuated by the denial of equal legal capacity, the CRPD Committee has gone as far to say that *any* assessments of decision-making ability which take account of a person’s disability are discriminatory. In General Comment 1, the CRPD Committee identifies three primary methods of assessment which it argues are routinely employed to deny legal capacity,^106^ all of which are enlivened in the context of insanity.

The first way is described as the ‘status approach’. This is where a diagnosis of disability forms the basis to deny legal capacity.^107^ Secondly, legal capacity is often denied on assessments that a decision is judged objectively irrational. This is described as the ‘outcome approach’. Finally, where a person’s decision-making capabilities are assessed as being deficient, then the CRPD Committee has said that the ‘functional approach’ provides the basis on which legal capacity is denied.^108^ Of the functional approach, General Comment 1 says:

It is often based on whether a person can understand the nature and consequences of a decision and/or whether he or she can use or weigh the relevant information. This approach is flawed for two key reasons: (a) it is discriminatorily applied to people with disabilities; and (b) it presumes to be able to accurately assess the inner-workings of the human mind and, when the person does not pass the assessment, it then denies him or her a core human right—the right to equal recognition before the law.^109^

Each of these assessments of mental capacity are operative in the context of the insanity defence, and form grounds to deny that the person was responsible for their actions. Firstly, the initial threshold for proof of insanity is evidence of the *status* of disability. That is, the person must be shown to have ‘a disease of the mind’ severe enough to impair the person’s thought processes to be able to be described as a ‘defect of reason’.^110^ In a court room, this requires evidence of a diagnosis by at least two or more registered physicians—usually psychiatrists^111^—and must be accepted by the finders of fact on the balance of probabilities. This *status* of disability operates as a threshold which, once crossed, compels further enquiry as to whether the defendant’s actions were driven by severe disability.^112^ The next step in establishing insanity is an *outcome* assessment of decision-making abilities. As an element of proof, the outcome to be established is the conduct element of the offence; the question is whether or not the person was able to reasonably foresee or rationally understand the consequences of their actions.^113^ This is a comparator question; in terms of the *actus reus*, the court is seeking to establish whether the defendant knew the *quality of the act* and, if they did, whether they knew it was *wrong*, compared with a defendant without the disability.^114^

Across jurisdictions, relevant laws require proof of both a diagnosis of a disability and the conduct element of the offence. However, these elements must be connected if insanity is to be made out. Thus, the final assessment by the finders of fact embodies the *functional* test, which requires evidence that the disability functioned to cause the person to engage in criminal conduct. An assessment will be made as to whether the person’s mental state was so severely impaired as to *deprive the defendant of reason*, so as to *not know* the nature and quality of the act or, if they did know, to not have *the ability to know* that what they were doing was wrong. Broadly, insanity applies where a diagnosis of mental disability is causative of the conduct element of the offence. From the perspective of criminal law, it is the nexus between the status, outcome and function of mental disability which provides justification for the special defence of insanity, its dispositions and treatment orders. While the Committee has not applied these tests specifically to insanity, the assessment of mental disability via the insanity doctrine is exactly the type of assessment that would breach Article 12 of the CRPD.

Also in General Comment 1, the CRPD Committee says

a person’s disability and/or decision-making skills are taken as legitimate grounds for denying his or her legal capacity and lowering his or her status as a person before the law. Article 12 does not permit such discriminatory denial of legal capacity, but, rather, requires that support be provided in the exercise of legal capacity.^115^

This oft-quoted passage is understood broadly to apply without restriction in any context.^116^ Read either in isolation or in conjunction with other guidance put out by the Committee, there is no scope to limit legal capacity, even in cases where a person’s mental disability was causative of a criminal act.^117^ On this analysis, it is understood that from the CRPD Committee’s perspective, all variations in decision-making processes are understood as value neutral.

Where decision-making processes are understood as qualitatively value neutral and decisions that are made are understood as an absolute expression of the person’s will, then respect for autonomy compels non-interference with decisions made by the person;^118^ failing to recognise a person’s decision as a valid expression of their assumed legitimate will and preferences is never compatible with the CRPD.^119^ Respecting all variations of decision making is part of the dignity of risk, and in upholding principles of equality and non-discrimination, decisions made by people with mental disabilities must be respected on the same basis as all others.^120^

The tensions in applying this interpretation of legal capacity in the context of criminal responsibility are best understood by returning to the case law. As the case of *Lau* demonstrates, the concept of criminal responsibility reflects a different understanding of the way that decision-making capabilities are understood to affect agency. In Lau’s case, reforming the insanity defence to make it disability neutral would likely mean that he would be called to answer for his crimes on the same basis as all others. Respect for his full legal agency would require criminal responsibility to be attributed, notwithstanding that his actions were animated by impairment. On the one hand, this approach might bring greater compliance with the CRPD. However, from the perspective of criminal justice, the discrimination perpetuated by treating Lau the same as all other defendants would be cause for alarm. Lau was unable to predict the deterioration in his mental state, he had little control over his actions and had no grip on reality. The harm that he caused to his family would presumably have been devastating to him. Attributing him responsible and punishing him accordingly seems unjust. From his wife and child’s perspective, this outcome is equally troubling; not only would the act be proved, but this would be accompanied by a finding that their husband and father had some intention^121^ to cause them harm. Instead of exculpation of guilt and a disposition focused on treatment and rehabilitation, Lau would be deemed criminally responsible.

However, if the CRPD Committee’s view of all variations of decision-making abilities as value neutral were to be adopted, Lau would be attributed responsibility for his actions and, by extension, for his state of mind. If the concept of *mens rea* was reformed to find Lau guilty, he would be held to normative standards of accountability, which he was functionally unable to meet.

This case is typical of the approach taken by the courts both in Hong Kong^122^ and more broadly across other common law jurisdictions.^123^ The Canadian case of *Attorney General of Ontario v G*^124^ further illustrates this point. In this case, the defendant experienced a single manic episode during which he was charged with twice sexually assaulting his then-wife, unlawfully confining her and harassment.^125^ His conduct which constituted the *actus reus* of the offence was not disputed. The defendant was acquitted on the basis that he was found not guilty by reason of mental disorder (NGMD). The medical evidence showed that his impaired mental state was causative of the crime, that it had been his first and only manic episode, that he was unable to reasonably have predicted that he would have such an episode, that he did not choose his mental state and that his behaviour was wholly inconsistent with his usual character. Once G received treatment, he went on to live 17 years as an upstanding member of the community, and the judge described his behaviour during that period as ‘spotless’.^126^ After G had been treated, the judge found that ‘there [wa]s no indication that he poses a risk to public safety’.^127^ Even G’s ex-wife—the victim of G’s conduct—corroborated his complete rehabilitation and supported the removal of the ongoing restrictions on his liberty.^128^

Certainly, the insanity defence worked to deny Lau’s and G’s legal capacity on the basis of an assessed absence of mental capacity. Recognising that G *had* been discriminated against by the ongoing restrictions on his liberty after his release, the Canadian Supreme Court made a declaration of invalidity of the relevant law, and immediately removed all remaining restrictions on G’s rights and liberties.^129^ That these discriminatory outcomes are problematic is not in issue.

However, the CRPD Committee’s interpretation of legal capacity offers little solution for reform. On the CRPD Committee’s interpretation, G’s decision would need to be respected as autonomous, and he should be attributed full criminal responsibility for the *actus reus* of his crimes. This would mean he should have been found guilty of the offence, and sentenced accordingly. Abolishing NGMD verdicts to instead attribute responsibility to G seems unduly punitive. Instead, under the current system, G spent two years being treated in a secure hospital.^130^

Some disability scholars have argued for the reform of *mens rea*^131^ and criminal responsibility^132^ to make these disability neutral. The *Lau* and *G* cases illustrate the way that the CRPD’s interpretation of legal capacity potentially compels a model of criminal law which is tied exclusively to a defendant’s subjective intent. Reform along these lines might exist as a sort of hybrid between what George P Fletcher^133^ and HLA Hart^134^ have described as a harm model of criminal law, which would render mental capacity irrelevant.^135^ Blame would be attributed for harm intended and caused, with little regard for the defendant’s mental functioning. In this sense, the criminal law would move back towards a strict liability approach to attributing guilt.^136^ Rather than bringing equality, this would reinforce a justice system premised on causal responsibility.^137^ It is surmised that abolishing insanity but maintaining a criminal justice system premised on capacity-responsibility may incline juries to convict a defendant for whom *mens rea* was proven, notwithstanding that their *mens rea* was wholly animated by mental disability. This is especially so where a crime was especially egregious or violent. For example, if, driven by his delusions, it was found that Lau had *intended* to stab and kill ghosts but instead stabbed his wife, the *mens rea* and *actus reus* for murder could be made out. An alternative defence considered in the literature of criminal legal theory is that of mistaken but delusion belief. As Claire Hogg’s analysis shows, this would leave juries with a similarly impossible task. The assessment would be one of whether the defendant’s response to the threat of demons was proportionate.^138^ Reforming the defence in either of these ways while maintaining the current criminal justice system would present juries with a stark choice; notwithstanding proof of the conduct element of a very serious crime, the options would be either a complete acquittal, with the defendant being released into the community, or conviction, where they would enter the corrections system.^139^ And while it is possible to envisage large-scale reform where treatment and support could be provided either within the community or in prison,^140^ these examples draw out the problems with the Committee’s approach. Abolition or reform of the insanity defence in these ways does nothing to address the inherent the ableism embedded within the institution of criminal justice. These alternatives do nothing to challenge the way that capacity-responsibility constructs the disabled mind as the other. Moreover, as argued by Linda Steele in the context of diversion, treatment would only be triggered once an egregious crime had been committed^141^—as the case of Matthew Choi (section 5A below) demonstrates.

The cases demonstrate the way that abolishing insanity, while failing to acknowledge the impact that disability can have on autonomy, is problematic. Recalling the presumption of capacity on which proof of *mens rea* is contingent, this category of defendants would be held to the same standards of capacity as other offenders for whom *mens rea* was proved. From a criminal justice perspective, there seems to be an inherent injustice in any failure to recognise the way that mental disability can animate *mens rea* and render it redundant.^142^ Indeed, a number of disability scholars have considered this issue, lending support to the contention that denying the impact of disability on responsibility would result in an ‘impoverished’^143^ criminal justice system. Criminal scholars have also considered this issue, broadly arguing that insanity itself is unnecessarily retained in the criminal justice system,^144^ and that alternative existing defences may better serve the purposes of the criminal justice system. Nonetheless, there is a consensus amongst criminal law scholars that consideration of the impact of mental disability is essential in the formulation of *mens rea*.^145^

There is no clear solution that will eliminate discrimination for people with mental disabilities within the existing limits of the current system. Instead, in line with a minority of disability scholars^146^ and criminal law scholars who work on the topic of insanity,^147^ I argue that reform which concentrates narrowly on doctrine is inadequate, and that any reform must take account of the impact of mental disability on reasons for acting.

The cases draw attention to an instinctive injustice in the Committee’s approach, which does not seem to grasp that the crime was not motivated by G’s or Lau’s authentic values, wishes or desires. Rendering the impact of mental disability on *mens rea* redundant would seem to corrode the criminal justice system’s moral component of restricting punishment only to those who are morally blameworthy. The criminal justice system’s deterrent function would similarly be weakened because the ‘undeterrable’ would be held to account to the same standards as offenders who had a fairer chance to avoid their actions. In fact, rather than conflating mental capacity with legal capacity, it would be the defendant’s intended conduct that was conflated with legal capacity. Notwithstanding the defendant’s mental capacity was wholly animated by disability, *mens rea* would be evidenced by an assessment of the defendant’s subjective mental state. A coincidence of the *actus reus* and *means rea* could be established even if the defendant’s state of mind was animated by temporary, but objectively false, beliefs, which were wholly inconsistent with the defendant’s authentic self. The concept of *mens rea* would be whittled away, to (somewhat ironically) single out a mental health crisis as the all-defining mental state of the person’s life. Rather than distinguishing the responsible from the non-responsible subject, this would essentially collapse the category of ‘mad’ into ‘bad’.^148^

I thus argue that there is a disjuncture between the CRPD Committee’s emphasis on providing robust recognition of the equal worth of, and support for, all variations of legal capacity and the reality of doing so. *G* and *Lau* are hard cases, for which the CRPD Committee’s interpretation of legal capacity does not offer a palatable solution. The defendant’s impaired decision-making capabilities were not value neutral, and their disabilities operated as a constraint on the exercise of their free will and authentic selves. Entrenching a universalised conception of legal capacity to hold G and Lau responsible for their actions would effectively hold them to a standard of capacity-responsibility that they were unable to meet by reason of an internal impairment.

## 5. Recognising Mental Impairment as an Internal Constraint on Autonomy: A Call for a More Nuanced Approach

### A. Mental Impairment as a Constraint on Autonomy

While the discrimination perpetuated by the insanity defence is unacceptable, it is the ableism that is embedded in the criminal justice system that ultimately requires challenge. In this section, I argue that the first step is to recognise that in some cases, mental disability is disabling. I draw from scholars who have argued that in doctrinally entrenching the spirit of a political movement, the CRPD does not satisfactorily grapple with the realities of impairment in many ‘hard cases’.^149^ Applying these ideas in the context of criminal responsibility, I make the case for moving beyond a narrow focus on legal capacity to target discrimination against people with mental disabilities by understanding its source. While I am unable to offer concrete solutions, what I do instead is identify various tools in the CRPD which move towards a more holistic understanding of the Convention as a whole.

Returning to another Hong Kong example provides insight of how mental impairment can constrain autonomy to manifest in tragic outcomes. This unreported case draws out some of the problems of failing to recognise mental impairment as inherently disabling.

In 2021, Matthew Choi Naam-sang fatally stabbed a taxi driver.^150^ The victim, a 48-year-old taxi driver, was a husband, a father of three and unknown to Choi. Choi was previously known to the authorities; in 2019, he had been arrested for possession of an offensive weapon and subsequently held in Siu Lam Psychiatric Centre.^151^ At that time, the media reported that the relevant charges were dropped when Choi’s uncle told prosecutors that Choi had schizophrenia, and that Choi had never had any intention to use the weapons in his possession. It was reported that Choi was subsequently released from Siu Lam Psychiatric Centre after an evaluation concluded that he did not have a mental disability.^152^

In the period between 2019 and the murder in 2021, Choi published a number of video clips on YouTube which had had tens of thousands of views, where he claimed that he was a ‘targeted individual’, linking his perceived victimisation to a conspiracy theory group in the United States.^153^ The YouTube clips create an impression that Choi had a confused sense of reality not shared by most other people. Assessed from any shared understanding of reality, Choi’s beliefs do appear delusional.

Choi consistently maintained that he was not mentally unwell. In the 2021 case, the prosecution applied for a psychiatric report, but Choi reportedly rejected the application, claiming that such report was not necessary.^154^ It is arguable that Choi may have lacked insight into his condition. The publicly available reports overwhelming suggest that Choi’s delusional beliefs compelled unpredictable and violent conduct, possibly even murder. The videos that Choi uploaded to YouTube certainly create an impression that his delusions prevented his thinking from conforming to normative standards.

Prior to the murder, Choi had a right to liberty and inclusion in the community on the same basis as all others. This right effectively trumped any risks that Choi was assessed to pose to the safety of the community. The law as it stands was unable to prevent what happened,^155^ which, viewed from all perspectives, seems far from satisfactory. But applying the Committee’s interpretation of the CRPD is extremely problematic. Viewed through the lens of the CRPD, Choi’s mental capacity would be required to be respected on the same basis as all others’. He had the right to maintain beliefs of persecution, even if those beliefs were not commensurate with objective standards of reasonableness or reality, and notwithstanding his overtly expressed intentions of violence. He had a right to reject coercive medical treatment. Applying Article 12, Choi would be understood to have the right to make decisions and mistakes, and to be held accountable for those decisions on the same basis as all others. This would be what the ‘dignity of risk’ required.

Choi’s case enables understanding of the consequences of embedding a conception of autonomy which embodies the CRPD Committee’s ‘hands-off’ approach to respect for decision making. At least in this case, respecting Choi’s decisions and actions as autonomous manifested in the murder of an innocent victim. Comparative hard cases have been considered in the context of civil commitment, but these arguments have not been fleshed out in the sphere of criminal law. I apply these arguments in the context of criminal responsibility, to draw out the implications of reform that embed this ‘thin’ conception of autonomy, which emphasises non-intervention with decision making.

### B. A More Nuanced Interpretation of Autonomy

Considering non-criminal cases where mental capacity is assessed to compromise decision-making, Camillia Kong writes that the Committee’s interpretation

does nothing to address the *internal* restraints and barriers that can restrict one’s freedom. Prioritising the internal domain of freedom ignores how, even there, unfreedom can occur. If unfreedom occurs *internally,* then it is question-begging as to why we would say external barriers somehow violate our freedom more than those internal barriers that directly impede the functioning of our motivations or knowledge of our authentic wishes.^156^

To put this another way, if a person’s will is ‘*structured* and *motivated*’^157^ by an internal impairment, then a decision cannot be said to be ‘autonomous in any rich sense’.^158^ As Choi’s case demonstrates, processes of decision making—the ‘functional’ aspect of decision making—*may* be *qualitative*. ‘Unfreedom’ can occur where a severe mental impairment operates as a restraint on free decision making.^159^ That is, if mental impairment wholly animates decision making and the person does not have the freedom to make choices or respond to reasons, autonomy is compromised by a constraining internal mental impairment.^160^ To push this analysis even further, giving effect to a person’s authentic self and values^161^ may compel intervention. In other words, not all unwanted third-party intrusions should be deemed illegitimate.^162^

Kong sees the problem with the CRPD Committee’s construction of autonomy as being the result of a conceptual confusion.^163^ She argues that the Committee conflates liberty with autonomy, but that these concepts are not the same. She writes that the Committee’s

odd formulation rests on some conceptual confusion about the precise relationship between positive and negative freedom … Interpretations of Article 12 seek to stress the illegitimacy of third-party intrusions, to emphasise how respect for an individual’s choices should outweigh welfarist, paternalistic concerns. But this articulation sits uneasily with the concept of positive liberty. In its most basic form, positive liberty emphasises that freedom is an expression of how the will is *structured* and *motivated*; it requires value judgments about one’s ends, desires, and preferences. According to this formulation, interventions by others can be justifiable. Or to put it differently, the premises of positive liberty do not immediately rule out others imposing decisions on you…^164^

To the extent that liberty is understood as an actor’s ability to act freely within the circumstances that they find themselves in, providing *conditions of liberty* may compel removing constraints to acting, *including* treating mental impairments which constitute internal constraints. A rich conception of autonomy requires not just the freedom to act and make choices, but also free conditions of acting.^165^ The question becomes whether or not the ‘choice’ to commit the criminal act was a free choice at all. In essence, the issue to be determined is whether or not a person was ‘a master of their own destiny’^166^ in the sense that within a given set of circumstances, the person is free to choose their course of conduct between the range of options available. Following Kong’s reasoning, support for autonomy may require interventions that remove internal barriers to liberty.

As the cases of Choi, *Lau*, *G*, *Winko* and *Swain* demonstrate, the insanity defence applies where a person is *not* a master of their own destiny in any real sense. Compelled by a mental disability, they are rendered unfree. In their impaired mental state, they had no fair chance to avoid their actions and were unable to respond to the world in a rational way. In other words, the defendant’s mental impairment constrained their ‘conditions of actions’.

In their theory of moral responsibility, John Fischer and Martin Ravizza describe this as an issue of ‘guidance control’.^167^ That is, individuals will be held responsible for acts which they undertook freely and for which they were able to exercise guidance control within the circumstances that they find themselves in.^168^ A compromised ability to understand or be reactive to their environment can limit a person’s opportunity to choose between alternative courses of acting and reacting. In their words, ‘it is *guidance control* that grounds moral responsibility for actions’.^169^ A person who lacks the ability to respond to external or internal stimuli lacks the guidance control necessary to be held morally responsible for their actions. On Fischer and Ravizza’s theory of moral responsibility, a person’s autonomy is reduced if they lack the ability to respond to reasons, and to this end, not all decisions are worthy of the same level of respect.^170^ Applying this reasoning in the context of criminal justice, the insanity defence applies in recognition that a person’s autonomy is reduced by the presence of mental disorder. Mental disorder which entirely animates decision making disables the ability to respond to reason, and blame is exculpated from wrongdoing. At the same time, diversion into psychiatric care is an intervention designed to remove internal barriers to liberty and a move towards restoring the defendant’s autonomy.

Notwithstanding this analysis, the discrimination that is perpetuated by the insanity defence and the ableism which is embedded within the institution of criminal justice is unacceptable. Accepting that something *must* be done to end disability discrimination, the burning question remains what can be done and how?

### C. Some Tools for Reform

Recognising both the doctrinal and philosophical problems with the Committee’s interpretation of autonomy, I argue for a more nuanced approach in terms of thinking about whether and how a psychosocial experience can affect a person’s agency,^171^ and further whether, in limited circumstances, this experience may compel intervention. I make this argument with great caution, recognising that, both historically and contemporaneously, responses to people with disabilities have been, and continue to be, inappropriate, degrading, coercive and even violent.^172^ I wish to emphasise that degrading and violent interventions are never acceptable. Still, this is not the same as saying that a person at risk, or a person who poses a significant risk to others, should not ever be ‘protected’ from or prevented from manifesting that risk.^173^ In the words of Jones and Shattell, I argue that overemphasising the right to make decisions potentially risks

“Sacrificing” the interests of individuals who have committed otherwise criminal acts, due to temporarily but profoundly altered beliefs or states, for the sake of a generalized and decontextualized “right” to legal capacity is, at a minimum, an advocacy goal that should be subject to the highest level of critical scrutiny and ethical reflexivity.^174^

Once again, civil commitment scholars offer a springboard to understanding how the CRPD might be interpreted differently, to move towards a richer notion of autonomy which may well turn out to be more consistent with giving effect to a person’s authentic self.

The Committee has said that giving effect to a person’s autonomy requires deference to a person’s expressed will and preferences.^175^ However, George Szmukler^176^ argues that, at times, ascertaining a person’s will and preferences is not always straightforward, especially where the person’s *will* and their *preferences* are in conflict. That is, at one time a person may express a short-term desire—or preference—to act in a particular way. However, this preference may conflict with the person’s long-term values, which Szmukler understands to be the person’s will and is reflective of their authentic self.^177^ Where there is any conflict, a person’s will should trump their preferences,^178^ even if this compels intervention. This hypothesis is understood when applied in the context of insanity when a person experiences delusions or a psychotic episode. As in the cases of *Lau* and *G*, delusions may animate a person’s preferences in the moment that a crime is committed. Delusions may compel the person to believe in that moment that their course of conduct is either necessary or justified. But those preferences may be in conflict with longer held authentic values of non-violence, love, care and goodwill towards their family, and a wish to abide by the civic norms set by criminal law. In such cases, deference to the defendant’s will would compel intervention.^179^

In a related vein, Kong and Ruck Keene take issue with what they see as the Committee’s overemphasis on demands to defer to a person’s *will and preferences* which seems to trump adherence to the person’s *rights* in the phrase ‘rights, will and preferences’.^180^ Emphasis on a person’s rights may well produce different outcomes, compared with focusing on their expressed will and preferences.^181^ For example, during a mental health crisis, adherence to a person’s expressed rejection of treatment may jeopardise their longer-term desire to live a long life. This conundrum is described by Peter Bartlett. He writes there may be a dilemma between a person’s rejection of treatment when their mental health is impaired, compared with being glad that they had treatment once their capacity has been restored.^182^ Elyn Saks argues that the challenge is both in making treatment available and in getting a person who is in the throes of a mental health crisis to want to be treated to improve their health.^183^ Having experienced psychosis, Anne Plumb^184^ writes that, in retrospect, she realised that her beliefs had been ridiculous, and that she had put herself at real risk.^185^ Subject to coercive medical interventions, what Plumb objected to was not being treated *per se*, but rather the manner in which the treatment was administered.^186^ It is suggested that shifting emphasis from support for legal capacity towards other rights in the CRPD, such as health,^187^ habilitation and rehabilitation,^188^ and community inclusion,^189^ may offer a way forward to reconceptualise what support for autonomy means.

This position is controversial as it conflicts with the Committee’s rejection of all unwanted coercive treatment as illegitimate.^190^ However, in the context of insanity, there may be real justification for taking a more nuanced approach to reconceptualise autonomy from a disability rights perspective. A conception of rich and robust autonomy can only challenge the ableist notion of criminal responsibility where there is recognition of both the inherent ableism embedded within the criminal justice system *and* the real limits that mental impairment can put on a person’s agency. Unfortunately, this article does not offer a simple solution as to how this can be achieved. Instead, what it calls for is a more holistic approach to supporting autonomy to ultimately bring about greater equality for people with disabilities who come into contact with the criminal justice system.

## 6. Conclusion

This article has argued that the CRPD Committee’s interpretation of legal capacity reinforces the structural inequality which is inherent in the criminal justice system’s organising norm of criminal responsibility. Ultimately, the ‘othering’ of mental disability will be reinforced by any ascription of criminal responsibility which does not take account of the way that mental disability can affect agency. In recognition of the way that mental impairment may operate as a constraint on autonomous functioning, the next steps towards achieving equality and inclusion for people with mental disabilities will be in working towards a more nuanced conception of legal capacity and perhaps a shift in emphasis in the way that rights are protected by the CRPD. Though the insanity defence cannot be left as it is, the target of reform needs to change; rather than focusing on doctrine, a nuanced understanding of the organising norms that shape those doctrines is called for. It is in this way that the notion of criminal responsibility may be drawn into question from a disability perspective, the ultimate aim being to challenge the structural injustice perpetuated against people with mental disabilities by the criminal justice system, its doctrines and processes.

## Footnotes

1
*HKSAR v Lau Kam Fai* (香 港 特 別 行 政 區 訴 劉 錦 輝) [2010] HKDC 633, DCCC 1029/2009 (20 April 2010).

2 ibid.

3 ibid para [16].

4 ibid para [5].

5 ibid para [12].

6 Criminal Procedure Ordinance, Cap 221 (CPO), s 74.

7 In Hong Kong, evidence of two or more psychiatrists is required for the Court to make an order that the accused be admitted to the ‘Correctional Services Department Psychiatric Centre or a mental hospital’; CPO, s76(2)(a). It was held in *HKSAR v Chow Kin Chung* [2007] HKCA 292, CACC 8/2007 (6 July 2007) para [12] that ‘if a defendant needs a hospital order and such an order is recommended, it is usually not desirable for the judge to ignore such order as the best sentencing option’. See also Jane Richards, ‘Discrimination against Defendants with Disabilities in the Hong Kong Criminal Justice System: Unfitness to Plead Rules, the Insanity Defence and Disposition Orders’ (2021) 51(3) HKLJ 875.

8 In Hong Kong, the exception is the Long-term Prison Sentences Review Ordinance, Cap 524.

9 Committee on the Rights of Persons with Disabilities, ‘Guidelines on the Right to Liberty and Security of Persons with Disabilities’ A/72/55 (2017) para [16].

10 See eg Tina Minkowitz, ‘Rethinking Criminal Responsibility from a Critical Disability Perspective: The Abolition of Insanity/Incapacity Acquittals and Unfitness to Plead, and Beyond’ (2014) 23(3) GLR 434; Amita Dhanda, ‘Universal Legal Capacity as a Universal Human Right’ in Michael Dudley, Derrick Silove and Fran Gale (eds), *Mental Health and Human Rights: Vision, Praxis, and Courage* (OUP 2012); Eilionóir Flynn and Anna Arstein-Kerslake, ‘Legislating Personhood: Realising the Right to Support in Exercising Legal Capacity’ (2014) 10(1) International Journal of Law in Context 81; Peter Bartlett, ‘The United Nations Convention on the Rights of Persons with Disabilities and Mental Health Law’ (2012) 75(5) MLR 752; Peter Bartlett, ‘A Mental Disorder of a Kind or Degree Warranting Confinement: Examining Justifications for Psychiatric Detention’ (2012) 16(6) International Journal of Human Rights 831; Peter Bartlett, ‘At the Interface between Paradigms: English Mental Capacity Law and the CRPD’ (2020) 11 Frontiers in Psychiatry 570735; Jillian Craigie, ‘Against a Singular Understanding of Legal Capacity: Criminal Responsibility and the Convention on the Rights of Persons with Disabilities’ (2015) 40 Int’l JL & Psychiatry 6; Jill Peay, ‘Mental Incapacity and Criminal Liability: Redrawing the Fault Lines?’ (2015) 40 Int’l JL & Psychiatry 25; Jill Peay, *Mental Health and Crime* (Routledge 2012); Meron Wondemaghen, ‘Testing Equality: Insanity, Treatment Refusal and the CRPD’ (2018) 25(2) Psychiatry, Psychology and Law 174; Law Commission of England and Wales (LCEW), ‘Criminal Liability: Insanity and Automatism, a Discussion Paper’ (2013); New Zealand Law Commission (NZLC), ‘Mental Impairment Decision-Making and the Insanity Defence’ (2010) Report 120; New South Wales Law Reform Commission (NSWLRC), ‘People with Cognitive and Mental Health Impairments in the Criminal Justice System: Criminal Responsibility and Consequences’ (2013) Report 138. Note that there are some disability scholars who consider there may be some advantages in maintaining an insanity defence. See eg John Dawson, ‘A Realistic Approach to Assessing Mental Health Laws’ Compliance with the UNCRPD’ (2015) 40 Int’l JL & Psychiatry 70; L Malatesti and others, ‘The Insanity Defence without Mental Illness? Some Considerations’ (2020) 71 Int’l JL & Psychiatry 101571.

11 Law Commission of England and Wales (n 10); NZLC (n 10).

12 See n 10.

13 It is noted that in his 2015 article, Christopher Slobogin adapts his ‘integrationist’ approach to the CRPD, the integrationist approach being explored in his 2006 book, which was published prior to the CRPD. Slobogin posits that his integrationist approach is akin to the Committee’s ‘disability neutral’ doctrines, thereby eliminating the need for specific mental disorder defences. Slobogin’s work is significant in this area, but it is noted that this article goes beyond his contribution. In citing Tina Minkowitz’s references to Slobogin, as Carole Petersen has noted, ‘it is doubtful that legislatures would ever adopt such vague defenses. It may be more prudent to propose reforms to the current defenses, providing stronger safeguards and more regular review of detention orders for defendants who are either deemed unfit for trial or relied upon disability based defenses’.See Carole J Petersen, ‘Addressing Violations of Human Rights in Forensic Psychiatric Institutions: Philosophical and Strategic Debates’ (2015) 109 Proceedings of the ASIL Annual Meeting 80, 82; Christopher Slobogin, ‘Eliminating Mental Disability as a Legal Criterion in Deprivation of Liberty Cases: The Impact of the Convention on the Rights of Persons with Disabilities on the Insanity Defense, Civil Commitment’ (2015) 40, Int’l JL & Psychiatry 36; Christopher Slobogin, *Minding Justice* (Harvard UP 2006); Minkowitz, ‘Rethinking Criminal Responsibility’ (n 10).

14 There is limited reference, if any, in the respective Law Commission reports to the CRPD (n 10).

15 See eg Craigie (n 10); Bartlett, ‘The United Nations Convention’ (n 10); Bartlett, ‘A Mental Disorder’ (n 10); Wondemaghen (n 10); Dawson (n 10).

16 On the challenge to criminal law doctrines from a disability perspective, Bartlett writes ‘A literature has begun to develop in this area … but it has tended to flow from disabilities law academics, not criminal law scholars. The latter need to be engaged if progress is to be made: this is not just about disability’. Bartlett, ‘At the Interface’ (n 10) 7.

17
*Daniel M’Naghten’s Case* [1843] UKHL J16.

18 J Bijlsma and others, ‘Legal Insanity and Risk: An International Perspective on the Justification of Indeterminate Preventive Commitment’ (2019) 66 Int’l JL & Psychiatry 101462.

19 ibid.

20 In her book, Kathryn Sikkink argues that although we may fall short of achieving full and robust notions of human rights protections, there is still value in continuing to strive for change, even if that change does not come all at once; see Kathryn Sikkink, *Evidence for Hope: Making Human Rights Work in the 21st Century* (Princeton UP 2017). See also Emilie Hafner-Burton, *Making Human Rights a Reality* (Princeton UP 2013). Both of these authors respond to criticisms that human rights are at a ‘twilight’ or ‘endtimes’. See further Eric A Posner, *The Twilight of Human Rights Law*, OUP 2014; Stephen Hopgood, *The Endtimes of Human Rights* (Cornell UP 2013).

21 For example, the LCEW report says, ‘We think that, because of the current interpretation of the *M’Naghten* test, a person with a disability within the meaning of section 6 of the Equality Act 2010 (which may include a person with a mental illness and/or learning difficulties) may be put at a disadvantage, as compared with a person without a disability. This can amount to unfair indirect discrimination contrary to section 19 of the Equality Act 2010’; Law Commission of England and Wales (n 10) para [1.74]. See further Law Commission reports across jurisdictions listed in n 10.

22 ibid para 1.106 cites *C* [2013] EWCA Crim 223, [1] and [20], [2013] All ER (D) 06 (Apr). See also the NZLC, which says that the purpose of the defence is ‘partly protecting some defendants, by shielding them from a criminal conviction … [and] protects the community, by ensuring that the defendant who would otherwise be entitled under normal principles of criminal liability to an acquittal can be detained’; NZLC (n 10) 4; the NSWLRC sees the purpose of the law as being to excuse criminal responsibility on the basis of impairment, while protecting the community through detention from those who pose a danger to themselves or others. More recent reforms shift the balance towards protection of the public from harm; NSWLRC (n 10) para [3.14]. See also Mental Health and Cognitive Impairment Forensic Provisions Act (NSW) 2020; NSW Parliament, Hansard Mental Health and Cognitive Impairment Forensic Provisions Bill 2020, Second Reading Speech, The Hon Bronnie Taylor, 16 June 2020; NSW Parliament, Hansard Mental Health and Cognitive Impairment Forensic Provisions Bill 2020, Second Reading Speech, Mr Mark Speakman, 3 June 2020.

23 Consistent with comparable jurisdictions, the NZLC concluded that ‘the overwhelming response was … the defence is workable, in spite of its flaws’; NZLC (n 10) 7. The LCEW found that the defence is well understood by jurors and that practitioners are able to make any necessary adjustments to ensure that it works as was intended because the defence fills a gap in the law where other outcomes are not appropriate; Law Commission of England and Wales (n 10). The NSWLRC found that the general consensus is that the defence works well in practice, despite the difficulties inherent and the correct outcome for defendants is able to be achieved; NSWLRC (n 10) para [3.19].

24 ‘Guidelines’ (n 9).

25 See further s 2B below.

26 The Hong Kong Solicitors’ Guide to Professional Conduct, vol 1, ch 5.01(4) says: ‘A solicitor cannot be retained by a prospective client who does not have mental capacity. There is a legal presumption of capacity unless the contrary is shown … If there is doubt about a prospective client’s mental capacity it may be advisable, where possible, to seek an opinion from the prospective client’s medical doctor.’

27 ibid ch 3.01, s 8. There are no equivalent provisions in the Hong Kong Bar Association’s Code of Conduct.

28 Former President of the Law Society of Hong Kong, Melissa Pang, reinforced this position. She said, ‘a legal representative having accepted instructions from a client … is under a duty to act in the best interests of the client and to provide a proper standard of service’. Melissa Pang, ‘President’s Message: Fundamental Rights in a Solicitor–Client Relationship’ *Hong Kong Lawyer* (March 2019) <www.hk-lawyer.org/content/fundamental-rights-solicitor-client-relationship> accessed 17 January 2024.

29 Canadian Charter of Rights and Freedoms, Constitution Act, 1982, ss 7, 9, 15.

30
*R v Swain* 1991 CanLII 104 (SCC), [1991] 1 SCR 933.

31 The Hong Kong Solicitors’ Guide to Professional Conduct, vol 1ch 1.01, Cap 159, sub leg H(c); Richards, ‘Discrimination against Defendants’ (n 7).

32
*HKSAR v Chow Kin Chung* (n 7).

33 ibid (emphasis added) [12] ; see also Richards, ‘Discrimination against Defendants’ (n 7).

34 Richards, ‘Discrimination against Defendants’ (n 7).

35
*Barker v Barker* 2017 ONSC 3397; *Barker v Barker* 2018 ONCA 255; *Barker v Barker* 2020 ONSC 3746;. For further analysis of the case, see Jane Richards, ‘An Incremental Approach to Filling Protection Gaps in Equality Rights for Persons with Disabilities’ (2021) 21(4) Human Rights Law Review 837.

36
*Barker v Barker* 2018 (n 35) 3, para [4].

37
*Winko v British Columbia (Forensic Psychiatric Institute)* [1999] 2 RCS.

38 Criminal Code of Canada, s 672.54 (Criminal Code); *R v Demers* 2004 SCC 46 (CanLII), [2004] 2 SCR 489.

39 CRPD, art 13; Richards, ‘Discrimination against Defendants’ (n 7).

40 CRPD art 12; Anna Arstein-Kerslake and others, ‘Human Rights and Unfitness to Plead: The Demands of the Convention on the Rights of Persons with Disabilities’ (2017) 17(3) Human Rights Law Review 399; Piers Gooding and Charles O’Mahoney, ‘Laws on Unfitness to Stand Trial and the UN Convention on the Rights of Persons with Disabilities: Comparing Reform in England, Wales, Northern Ireland and Australia’ (2016) 44 International Journal of Human Rights 122; Piers Gooding and others, ‘Unfitness to Stand Trial and the Indefinite Detention of Persons with Disabilities in Australia: Human Rights Challenges and Proposals for Change’ (2017) 40 MULR 816.

41 CRPD, art 14; Catalina Devandas-Aguilar, Special Rapporteur on the Rights of Persons with Disabilities, ‘Report of the Special Rapporteur on the Rights of Persons with Disabilities’ (Human Rights Council 2019) A/HRC/40/54; Minkowitz, ‘Rethinking Criminal Responsibility’ (n 10).

42 CRPD, art 17; Minkowitz, ‘Rethinking Criminal Responsibility’ (n 10).

43 CRPD, art 16; *Barker v Barker* 2017 (n 35); *Barker v Barker* 2018 (n 35); *Barker v Barker* 2020 (n 35); Richards, ‘An Incremental Approach’ (n 35).

44 CRPD, art 15; Minkowitz, ‘Rethinking Criminal Responsibility’ (n 10).

45 Committee on the Convention on the Rights of Persons with Disabilities, ‘General Comment No 1, Article 12: Equal Recognition Before the Law’ (2014) CRPD/C/GC/1, para [5] (GC 1).

46 ibid para [5].

47 ibid para [8]. On the CRPD Committee’s interpretation, legal capacity is indispensable for the exercise of civil, political, economic, social and cultural rights; ibid paras [5], [8], [25]. See also Anna Arstein-Kerslake and Eilionóir Flynn, ‘The General Comment on Article 12 of the Convention on the Rights of Persons with Disabilities: A Roadmap for Equality Before the Law’ (2016) 20 International Journal of Human Rights 471; Anna Arstein-Kerslake, *Restoring Voice to People with Cognitive Disabilities: Realizing the Right to Equal Recognition Before the Law* (CUP 2017); Dhanda, ‘Universal Legal Capacity’ (n 10); Melvyn Freeman and others, ‘Reversing Hard Won Victories in the Name of Human Rights: A Critique of the General Comment on Article 12 of the UN Convention on the Rights of Persons with Disabilities’ (2015) 2 Lancet Psychiatry 8440850; Tina Minkowitz, ‘Norms and Implementation of CRPD Article 12’ (2010) <https://papers.ssrn.com/sol3/papers.cfm?abstract_id=2037452>; Rosemary Kayess and Phillip French, ‘Out of Darkness into Light? Introducing the Convention on the Rights of Persons with Disabilities’ (2008) 8(1) Human Rights Law Review 1; Gerard Quinn, ‘The United Nations Convention on the Rights of Persons with Disabilities: Toward a New International Politics of Disability’ (2009) Texas Journal on Civil Liberties & Civil Rights 33.

48 CRPD, art 12(2).

49 GC 1 (n 45) para [14] (emphasis added).

50 GC 1 says, ‘The concept of mental capacity is highly controversial in and of itself. Mental capacity is not, as is commonly presented, an objective, scientific and naturally occurring phenomenon. Mental capacity is contingent on social and political contexts, as are the disciplines, professions and practices which play a dominant role in assessing mental capacity”; ibid para [15]; Clíona de Bhailís and Eilionóir Flynn, ‘Recognising Legal Capacity: Commentary and Analysis of Article 12 CRPD’ (2017) 13(1) International Journal of Law in Context 6.

51 GC 1 (n 45) para [4]; Amita Dhanda, ‘Legal Capacity in the Disability Rights Convention: Stranglehold of the Past or Lodestar for the Future’ (2006) 34 Syracuse J Int’l L & Com 429.

52 GC 1 (n 45) para [13].

53 ibid para [13]; Arstein-Kerslake and Flynn, ‘The General Comment’ (n 47).

54 GC 1 (n 45) para [14].

55 ‘Guidelines’ (n 9) para [14]; Arstein-Kerslake (n 47).

56 ‘Guidelines’ (n 9).

57 This interpretation of legal capacity is not shared by all other UN bodies. For example, compare Devandas-Aguilar (n 41); Human Rights Council, ‘10th Session, Report of the Special Rapporteur on the Right of Everyone to the Enjoyment of the Highest Attainable Standard of Physical and Mental Health’ (UN General Assembly 2020) A/HRC/44/48; Human Rights Council, ‘Twenty-second Session, Report of the Special Rapporteur on Torture and Other Cruel, Inhumane or Degrading Treatment or Punishment, Juan E. Mendez’ (UN General Assembly 2013) A/HRC/22/53; Special Rapporteur on the Right of Everyone to the Highest Attainable Standard of Health, ‘Right of Everyone to the Highest Attainable Standard of Physical and Mental Health’ (UN General Assembly 2009) A/64/272; Dainius Pūras, Special Rapporteur on the Right of Everyone to the Enjoyment of the Highest Attainable Standard of Physical and Mental Health, ‘Report of the Special Rapporteur on the Right of Everyone to the Enjoyment of the Highest Attainable Standard of Physical and Mental Health’ (Human Rights Council 2020) A/HRV/44/48; Human Rights Council, ‘Report of the Special Rapporteur on the Right of Everyone to the Enjoyment of the Highest Attainable Standard of Physical and Mental Health’ (UN General Assembly 2018) A/HRC/38/36; William A Schabas*, UN International Covenant on Civil and Political Rights: Nowak’s CCPR Commentary* (3rd edn, NP Engel 2019). Still, there is an argument that significant deference should be shown to how disability rights are interpreted by the Committee, given that this is within the scope of its mandate. I am grateful to Peter Bartlett for making this point to me.

58 George P Fletcher, *Rethinking Criminal Law* (OUP 2000) ch 6; Nicola Lacey, *In Search of Criminal Responsibility: Ideas, Interests, and Institutions* (OUP 2016) 30 writes ‘So long as we are confident that the defendant has the capacities of a reasonable person, this fair opportunity view is perfectly consistent with the moral intuition underlying the capacity principle of criminal responsibilty’.

59 Claire Hogg and JJ Child, ‘Not Guilty by Reasons other than Insanity’ in AP Simester (ed), *Modern Criminal Law: Essays in Honour of GR Sullivan* (Hart Publishing 2024) 156.

60
*R v Layton* (1849) 4 Cox 149; *M’Naghten* (n 17).

61 Lindsay Farmer, *Making the Modern Criminal Law* (OUP 2016) 168.

62 Lacey (n 58) 36; Alan W Norrie, *Law, Ideology and Punishment* (Kluwer Academic Publishers 1991) 162 writes ‘the emergence of the liberal ideal of criminal justice with its emphasis on individual responsibility is closely connected with the development of modern theories of punishment’.

63 Farmer (n 61).

64 ibid 163 (footnotes omitted).

65 Lacey (n 58) 27.

66 ibid 27 (footnotes omitted).

67 Arlie Loughnan, *Manifest Madness: Mental Incapacity in Criminal Law* (OUP 2012) ch 1.

68 David Brown and others, *Criminal Laws: Materials and Commentary on Criminal Law and Processes* (4th edn, The Federation Press 2006) 330.

69 This is the principle of the contemporaneity of *actus reus* and *mens rea; Jakeman* (1983) 76 Cr App R 223; *Styles* [2015] EWCA Crim 1619; *Kaitamaki v The Queen* [1985] AC 147; *Fagan v Metropolitan Police Commissioner* [1969] 1 QB 439; *Thabo Meli v The Queen* [1954] 1 All ER 373; *Church* [1966] 1 QB 59.

70
*R v Sargeant* (1974) 60 Cr App R 74; Simon NM Young, ‘Sentencing’ in Eric Wing Hong Chui and T Wing Lo (eds), *Understanding Criminal Justice in Hong Kong* (2nd edn, Routledge 201) 268; Young notes that conciliation is now a recognised principle in many places, but not in Hong Kong. See also Fletcher (n 58); HLA Hart, *The Concept of Law* (OUP 2012).

71 ‘Disease of the mind’ is the language of *M’Naghten* (n 17). Contemporaneously, the language adopted in Hong Kong for proof of insanity is that the person is ‘under a disability’, CPO, ss 2, 75A. The language in the Criminal Code, s 16(1) is that of ‘mental disorder’. Note there are some jurisdictions which are an exception to this generalisation. In Norway, the mere presence of mental disorder satisfies the criminal incapacity standard: Norway Penal Code, s 20; Michael S Moore, ‘The Quest for a Responsible Responsibility Test: Norwegian Insanity Law after Breivik’ (2014) 9(4) Criminal Law and Philosophy 645; Linda Gröning, ’Criminal Insanity in Norwegian Law’ in Ronnie Mackay and Warren Brookbanks (eds), *The Insanity Defence: International and Comparative Perspectives* (OUP 2022) <https://doi.org/10.1093/oso/9780198854944.003.0013> accessed 19 December 2023. Sweden does not have an insanity defence; Susana Radovic, Gerben Meynen and Tova Bennet, ‘Introducing a Standard of Legal Insanity: The Case of Sweden Compared to the Netherlands’ (2015) 40 Int’l JL & Psychiatry 43; Piers Gooding and Tova Bennet, ‘The Abolition of the Insanity Defence in Sweden and the United Nations Convention on the Rights of Persons with Disabilities: Human Rights Brinkmanship or Evidence it Does Not Work?’ (2018) 21 New Criminal Law Review 141.

72 Hogg and Child (n 59).

73
*M’Naghten* (n 17).

74 CPO, s 75A; Criminal Code, s 16.

75 Loughnan (n 67) 55.

76 ibid.

77 Malatesti and others (n 10).

78 Loughnan (n 67).

79 Bijlsma and others (n 18). In *Winko* (n 37), the court held that though the relevant legislative provisions did not create a presumption of dangerousness, the balance must be struck in favour of public safety, and the defendant is not to be released from any sort of supervision order ‘until it is established that he or she is not a significant threat to the safety’ (632).

80
*Winko* (n 37); *HKSAR v Tsang Man Wai, Raymond* (曾文緯) [2017] 4 HKC 419, [2017] HKCU 1474; *Pither* (1979) Cr App R (S) 209; Attorney General’s Reference No 32 of 1996 (Whittaker) [1997] 1 Cr App R(S) 261; *R v Hodgson* (1968) Cr App R 113.

81 Michel Foucault, *Madness and Civilization* (Vintage 1965) writes of the ‘mad’ presenting a literally and figurative threat to normative order.

82 ibid.

83 Farmer (n 61) 20.

84 ibid.

85 Lacey (n 58) 37 writes ‘criminal law exhibits an inclusionary versus an exclusionary temper, and for how far it is seen as addressing free and equal subjects as opposed to managing a threat posed by particular categories of subject’.

86 Loughnan (n 67) 24.

87 Loughnan describes this as the ‘terrain of mental incapacity in criminal law’; ibid ch 1.

88 ibid; in Loughnan’s words, ‘By constructing the non-responsible subject as abnormal, the ‘normal’ individual becomes a responsible legal subject, one to whom ordinary principles of responsibility, liability, and punishment apply’ (34).

89 Lucy Series, *Deprivations of Liberty in the Shadows of The Institution* (Bristol UP 2022); Gregory Zilboorg and George W Henry, *A History of Medical Psychology* (WW Norton 1941); Franz G Alexander and Sheldon T Selesnick, *The History of Psychiatry: An Evaluation of Psychiatric Thought and Practice from Prehistoric Times to the Present* (George Allen and Unwin 1966); Christina Vanja, ‘Madhouses, Children’s Wards, and Clinics: The Development of Insane Asylums in Germany’ in N Finzsch and R Jütte (eds), *Institutions of Confinement: Hospitals, Asylums, and Prisons* (CUP 1996) 117.

90 Iris Marion Young, *Responsibility for Justice* (OUP 2011).

91 Lacey (n 58) writes ‘While we think of ourselves as a society of equal opportunities and social inclusion, our criminal justice system serves to legitimize the impact of structural inequalities based on social cleavages, such as race, by labelling manifestations of allegedly dangerous difference as criminal’ (173).

92 Loughnan (n 67) 7.

93 Fletcher (n 58).

94 Linda Steele, *Disability, Criminal Justice and Law: Reconsidering Court Diversion* (Routledge 2020).

95 ibid introduction.

96 Malatesti and others (n 10); Gerben Meynen, ‘Exploring the Similarities and Differences between Medical Assessments of Competence and Criminal Responsibility’ (2009) 12(4) Medicine, Health Care, and Philosophy 443. Robinson and Darley write ‘Criminal law’s influence comes from being a societal mechanism by which the force of social norms is realized and by which the force of internal principles is strengthened’; Paul H Robinson and John M Darley, ‘The Utility of Desert’ (1996) 91(2) Northwestern University Law Review 453, 471.

97 Lacey (n 58) 84.

98 Steel (n 94).

99 ‘Guidelines’ (n 9) para [16].

100 ibid.

101 Steele (n 94) ch 7.

102 ibid 65; Liat Ben-Moshe, *Decarcerating Disability: Deinstituionalization and Prison Abolition* (University of Minnesota Press 2020) 10.

103 GC 1 (n 45) para [13].

104 ibid.

105 Arstein-Kerslake (n 47).

106 GC 1 (n 45) para [15].

107 ibid para [15].

108 ibid para [15].

109 ibid para [15].

110 Compare CPO, s 75A and Criminal Code, s 16.

111 CPO, s 76(2); Mental Health Ordinance, Cap 136; Criminal Code, s 672.39; NSW, Australia, is an exception and medical evidence is not strictly required; *R v Lucas* [1970] HCA 14, (1970) 120 CLR 171; *R v Ilie Istudor* [2016] NSWDC 1. However, in practice, it is usual to call for evidence from two psychiatrists or psychologists; Mental Health Act (NSW) 2007; Mental Health and Cognitive Impairment Forensic Provisions Act (NSW) 2020.

112 Sweden presents an exception to this (n 71).

113 The language of the *M’Naghten* test is whether the person understood the ‘nature and quality’ of the conduct element of the offence or knew that the conduct ‘was wrong’.

114 On the issue of establishing a comparator, see *Purvis v New South Wales (Department of Education and Training)* [2503] HCA 62; 217 CLR 92; 202 ALR 133; 78 ALJR 1.

115 GC 1 (n 45) para [15].

116 Flynn and Arstein-Kerslake (n 10).

117 ‘Guidelines’ (n 9) para [16]. Note that the drafters of the CRPD expressly rejected inclusion of the MI Principles, which make provision for non-consensual treatment (Principle 11) and hospital orders for a finding of insanity (art 20); General Assembly Resolution 46/119, Principles for the Protection of Persons with Mental Illness and the Improvement of Mental Health Care (1991); Janet E Lord, ‘Preamble’ in Ilias Bantekas, Michael Ashley Stein and Dimitris Anastasiou (eds), *The Convention on the Rights of Persons with Disabilities: A Commentary* (OUP 2018) 5.

118 Flynn and Arstein-Kerslake (n 10) 91.

119 GC 1 (n 45) para [17].

120 Dhanda, ‘Legal Capacity’ (n 51); GC 1 (n 45) para [22].

121 See further the discussion on alternatives conceptions of *mens rea* below.

122
*HKSAR v Liu Zhengyan* (香 港 特 別 行 政 區 訴 劉 正 彥) [2018] HKDC 1138, DCCC 1143/2017 (7 September 2018); *HKSAR v Ho Kui-yao* (香 港 特 別 行 政 區 訴 何 巨 堯) [2012] HKDC 1347, DCCC 518/2012 (4 October 2012); *HKSAR v Hung Weicong* (香 港 特 別 行 政 區 訴 洪 維 聰) [2013] HKDC 1375, DCCC 345/2013 [2013] (19 September 2013); *HKSAR v Wan Pak Sing* [2004] HKCA 163, [2004] 3 HKC 283, CACC 535/2003 (28 May 2004); *HKSAR v Yu Sau* [2010] HKCFI 2128, HCCC 58/2009 (20 July 2010); *HKSAR v Ke Xiangchao* (香 港 特 別 行 政 區 訴 柯 向 朝) [2016] HKDC 137, DCCC 245/2015 (15 February 2016). Note, cases were read in English translation from the original traditional Chinese.

123 In Canada see *Swain* (n 30); *Winko* (n 37).

124
*Attorney General of Ontario v G* 2020 SCC 38.

125 ibid para [7].

126 ibid paras [5], [182].

127 ibid para [182].

128
*Attorney General of Ontario v G*, ‘Respondent’s Response to Application for Leave to Appeal’, Court file no 38585.

129
*Attorney General of Ontario v G* (n 124).

130 It is not suggested that people in forensic hospitals are ‘better off’, nor that forensic care is a satisfactory and legitimate solution. This case is included to show that in some cases, treatment may be the most appropriate response within the limited range of options which currently exist.

131 Flynn and Arstein-Kerslake (n 10) 88.

132 Minkowitz, ‘Rethinking Criminal Responsibility’ (n 10).

133 Fletcher (n 58).

134 Hart (n 70).

135 Bartlett, ‘A Mental Disorder’ (n 10).

136 Dawson (n 10) 73.

137 Lacey (n 58) ch 5; Fletcher (n 58).

138 Claire Hogg, ‘Reasonable Expectations for “Unreasonable” People: Reframing Objective Standards in Criminal Law’ (in press). Hogg writes ‘Courts have typically taken the view that any attempt to allow the presence of a mental condition—particularly and especially a mental condition characterised by delusions and/or hallucinations—to modify a reasonableness standard will result in nonsense; the “reasonable schizophrenic” is perceived as less a coherent standard than a contradiction in terms’ (1) (footnotes omitted).

139 Thanks to Prof Simon NM Young for making this point to me.

140 Mad studies scholarship offers alternatives to the current system, including the abolition of prisons and psychiatry. See Ben-Moshe (n 102); Liat Ben-Moshe, Chris C Chapman and Allison C Carey, *Disability Incarcerated: Imprisonment and Disability in the United States and Canada* (Palgrave Macmillan 2014); Tina Minkowitz, ‘CRPD Advocacy by the World Network of Users and Survivors of Psychiatry: The Emergence of an User/Survivor Perspective in Human Rights (August 14, 2012) <https://ssrn.com/abstract=2326668> or <http://dx.doi.org/10.2139/ssrn.2326668>.

141 Steele (n 94).

142 Malatesti and others (n 10).

143 Dawson (n 10) 73.

144 Some criminal law scholars offer alternative means for abolishing insanity, but these are not from a disability rights perspective; Claire Hogg, ‘The Insanity Defence: An Argument for Abolition’ (2015) 79(4) JCL 250; Hogg, ‘Reasonable Expectations’ (n 138); Christopher Slobogin, ‘Guilty but Mentally Ill Verdict: An Idea Whose Time Should Not Have Come’ (1984) 53 Geo Wash L Rev 494; Christopher Slobogin, ‘An End to Insanity: Recasting the Role of Mental Disability in Criminal Cases’(2000) 86 Va L Rev 1199; Slobogin, *Minding Justice* (n 13).

145 Further consideration of reformulating *mens rea* from a disability perspective from a disability rights perspective is worthy of its own study and goes beyond the scope of this article.

146 Bartlett, ‘A Mental Disorder’ (n 10); Dawson (n 10); Craigie (n 10); Peay (all n 10).

147 Hogg, ‘Reasonable Expectations’ (n 138); Slobogin, ‘An End to Insanity’ (n 144).

148 Loughnan writes ‘the construction of “madness” at the point of intersection with crime shares features with character-based conceptions of responsibility: that is, as it is constructed as dispositional, “madness” for criminal law purposes is character-like’; Loughnan (n 67) 51, see further ch 3. See also Human Rights Council, A/HRC/38/36 (n 57) para [52].

149 Tom Shakespeare, *Disability Rights and Wrongs Revisited* (Routledge 2006); Eva Feder Kittay with Leo Kittay ‘On the Expressivity and Ethic of Selective Abortion for Disability: Conversations with My Son’ in Erik Parens and Adrienne Asch (eds), *Prenatal Testing and Disability Rights* (Georgetown UP 2007).

150 ‘Man in Court on Taxi Driver Murder Charge’ *RTHK* (16 October 2021) <https://news.rthk.hk/rthk/en/component/k2/1615503-20211016.html> accessed 6 June 2022.

151 Tom Grundy, ‘“Alarm Bells Rang”: How a Lamma Island Resident Alerted Police after Realising “Drinking Partner” Was Murder Suspect On-the-run’ *HKFP* (14 October 2021) <https://hongkongfp.com/2021/10/14/alarm-bells-rang-how-a-lamma-island-resident-alerted-police-after-realising-drinking-partner-was-murder-suspect-on-the-run/> accessed 6 June 2022; Dimsum Daily, ‘Murder Suspect Matthew Choi Who Killed Taxi Driver Arrested on Lamma Island (Updated: 10.20pm)’ *Coconuts Hong Kong* (13 October 2021) <www.dimsumdaily.hk/murder-suspect-who-killed-taxi-driver-arrested-on-lamma-island/> accessed 6 June 2022.

152 ‘Man Who Murders Cabbie Was Psych Patient, Once Threatened Mass Shooting’ *Coconuts Hong Kong* (13 October 2021 <https://coconuts.co/hongkong/news/man-who-murdered-taxi-driver-was-psych-patient-once-threatened-mass-shooting/> accessed 6 June 2022.

153 Matthew Choi, ‘Matthew Choi TI’ (YouTube 17 March 2019) <www.youtube.com/watch?v=WsBgCAkZu2c;TLOKE 57> accessed 6 June 2022; ‘Matthew Choi Testimony’ (YouTube 15 October 2021) <www.youtube.com/watch?v=pwKdgefFbe4> accessed 6 June 2022.

154 ‘Taxi Driver Murderer Matthew Choi Remanded at Siu Lam Psychiatric Centre till 29th Oct’ *Dimsum Daily* (16 October 2021) <www.dimsumdaily.hk/taxi-driver-murderer-matthew-choi-remanded-at-siu-lam-psychiatric-centre-till-29th-oct/> accessed 6 June 2022.

155 Thank you to Prof Simon NM Young for making this point to me.

156 Camillia Kong, *Mental Capacity in Relationship: Decision-Making, Dialogue and Autonomy* (CUP 2017) 36 (emphasis in original, footnotes omitted).

157 ibid 35 (emphasis in original).

158 Jonathan Herring, *Law and the Relational Self* (CUP 2019) 120.

159 Kong (n 156).

160 ibid.

161 ibid; Camillia Kong and Alex Ruck Keene, *Overcoming Challenges in the Mental Capacity Act 2005* (Jessica Kingsley Publishers 2018).

162 Kong (n 156) 36.

163 ibid.

164 ibid 35 (emphasis in original).

165 ibid.

166 ibid. Scott Veitch writes ‘“Autonomy” means literally “self-rule”’; Scott Veitch, *Obligations: New Trajectories in Law* (Routledge 2021) 36.

167 John Martin Fischer and Mark Ravizza, *Responsibility and Control* (CUP 2000).

168 ibid 54.

169 ibid 54 (emphasis in original).

170 Herring (n 158) writes ‘In short, not all autonomous decisions deserve the same level of protection’ (118).

171 Anne Plumb, ‘UN Convention on the Rights of Persons with Disabilities: Out of the Frying Pan Into the Fire? Mental Health Service Users and Survivors Aligning with the Disability Movement’ in Helen Spandler, Jill Anderson and Bob Sapey (eds), *Madness, Distress and the Politics of Disablement* (Policy Press 2015) 192.

172 Series (n 89); Vanja (n 89); Zilboorg and Henry (n 89); Alexander and Selesnick (n 89).

173 Plumb (n 171) 191. The language of ‘protection’ may be interpreted as reinforcing outdated attitudes of paternalism and benevolence. However, I follow Plumb’s scholarship, arguing that a person may require protection from engaging in risky behaviour.

174 Nev Jones and Mona Shattell, ‘Beyond Easy Answers: Facing the Entanglements of Violence and Psychosis’ (2014) 35 (10) Issues in Mental Health Nursing 809, 810.

175 GC 1 (n 45).

176 George Szmukler, *Men in White Coats: Treatment under Coercion* (OUP 2017); George Szmukler, ‘“Capacity”, “best interests”, “will and preferences” and the UN Convention on the Rights of Persons with Disabilities’ (2019) 18(1) World Psychiatry 34.

177 Szmukler, ‘“Capacity”’ (n 176).

178 ibid.

179 See eg Choi’s case, above.

180 Kong and Ruck Keene (n 161). See also *PBU & NJE v Mental Health Tribunal* [2018] VSC 564 (1 November 2018); Piers Gooding, *A New Era for Mental Health Law and Policy: Supported Decision-Making and the UN Convention on the Rights of Persons with Disabilities* (CUP 2018) 143.

181 Kong (n 156); Kong and Ruck Keene (n 161); Peter Bartlett, ‘Will and Preferences in the Overall CRPD Project’ (2019) 18(1) World Psychiatry 48. There may be conflicts between prioritising a person’s expressed will and preferences (art 12) over the right to health (art 25) if refusal of treatment will otherwise compromise health. Similarly, the right to personal integrity (art 17) may conflict with a right to rehabilitation (art 26).

182 Bartlett, ‘At the Interface’ (n 10).

183 New Books in Law, ‘Elyn Saks, “Mental Health: Policies, Laws and Attitudes”’ (New Books Network 2021) <https://newbooksnetwork.com/elyn-saks-mental-health-policies-laws-and-attitudes-open-agenda-2021> accessed 2 February 2022; Dilip V Jeste and Elyn Saks, ‘Capacity to Consent to or Refuse Treatment and/or Research: Theoretical Considerations’ (2006) 24(4) Behavioral Sciences & the Law 411.

184 Plumb (n 171).

185 ibid 188.

186 ibid.

187 CRPD, art 25.

188 CRPD, art 26.

189 CRPD, art 19.

190 Minkowitz, ‘CRPD Advocacy’ (n 140).

